# Ischemic Tolerance—A Way to Reduce the Extent of Ischemia–Reperfusion Damage

**DOI:** 10.3390/cells12060884

**Published:** 2023-03-13

**Authors:** Rastislav Burda, Jozef Burda, Radoslav Morochovič

**Affiliations:** 1 Department of Trauma Surgery, Faculty of Medicine, Pavol Jozef Šafárik University in Košice, Rastislavova 43, 040 01 Košice, Slovakia; 2 Department of Trauma Surgery, Louis Pasteur University Hospital, Rastislavova 43, 040 01 Košice, Slovakia; 3 Institute of Neurobiology, Slovak Academy of Sciences, 040 01 Košice, Slovakia

**Keywords:** ischemia–reperfusion injury, ischemic tolerance, preconditioning, postconditioning

## Abstract

Individual tissues have significantly different resistance to ischemia–reperfusion damage. There is still no adequate treatment for the consequences of ischemia–reperfusion damage. By utilizing ischemic tolerance, it is possible to achieve a significant reduction in the extent of the cell damage due to ischemia–reperfusion injury. Since ischemia–reperfusion damage usually occurs unexpectedly, the use of preconditioning is extremely limited. In contrast, postconditioning has wider possibilities for use in practice. In both cases, the activation of ischemic tolerance can also be achieved by the application of sublethal stress on a remote organ. Despite very encouraging and successful results in animal experiments, the clinical results have been disappointing so far. To avoid the factors that prevent the activation of ischemic tolerance, the solution has been to use blood plasma containing tolerance effectors. This plasma is taken from healthy donors in which, after exposure to two sublethal stresses within 48 h, effectors of ischemic tolerance occur in the plasma. Application of this activated plasma to recipient animals after the end of lethal ischemia prevents cell death and significantly reduces the consequences of ischemia–reperfusion damage. Until there is a clear chemical identification of the end products of ischemic tolerance, the simplest way of enhancing ischemic tolerance will be the preparation of activated plasma from young healthy donors with the possibility of its immediate use in recipients during the initial treatment.

## 1. Ischemia–Reperfusion (IR) Injury

The term ischemia–reperfusion injury refers to a condition in which tissue ischemia occurs with insufficient oxygen supply for the tissue’s current metabolic needs, followed by the successful restoration of blood flow.

Ischemia with reperfusion initiates a wide complex of changes of an inflammatory nature that can worsen local conditions and also lead to dysfunction of a distant organ or the entire organism.

Ischemia occurs in acute arterial occlusions (an ischemic cerebrovascular accident, myocardial infarction, and ischemia of a limb), where reperfusion subsequently occurs as part of treatment (thrombolytic treatment, angioplasty, surgical revascularization, and operations with tourniquet application) [1]. Moreover, common surgical procedures (organ transplantation, transfer of free flaps, cardiopulmonary bypass, vascular operations, or polytrauma patients) lead to ischemia–reperfusion damage [2].

In transplant recipients, attention has been focused on post-transplant patient management in the past, with an obvious emphasis on immunosuppression, but “the biggest hit” to the donor organ is encountered during the process of donation and reperfusion at the time of transplantation. The problem is in the uncontrolled ROS (reactive oxygen species) formation during the reperfusion phase, which leads to mitochondrial disfunction (opening of mPTP/mitochondrial transition pores/and the release of DAMPs/damage-associated molecular patterns/in the intra- and extracellular space) [3,4,5,6].

**Figure 1:**
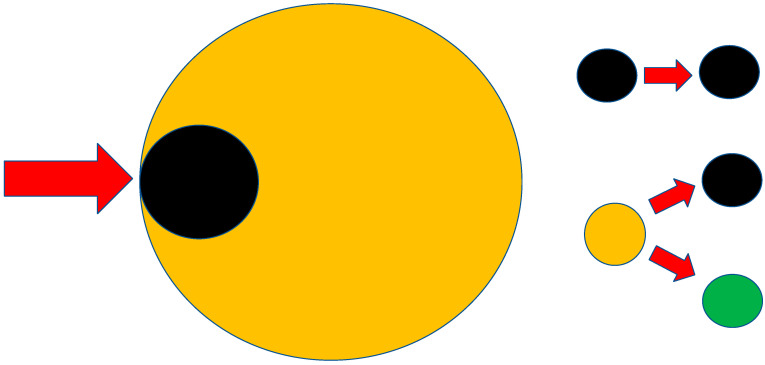
Schematic illustration of the effect of IR damage. As a result of ischemia, some of the cells are primarily irreversibly damaged; they can no longer be influenced therapeutically. Reversibly damaged cells can be influenced. The next approach can lead to their recovery or to irreversible necrosis. Explanations: the yellow circle with the black one inside represents all cells that experienced ischemia, the yellow circle represents the cells with reversible damage, the black circle represents the dead cells (irreversible damage), and the green circle represents the surviving viable cells.

In cardiomyocyte tissue cultures, 4 h of simulated ischemia resulted in the death of up to 17% of the cells, but the subsequent reperfusion resulted in the death of up to 73% of the ischemia-affected cells in the experiment [7]. More cells died because of the subsequent reperfusion than from the ischemia alone. Some of the cells of the ischemic tissue remain irreversibly damaged as a result of the action of ischemia. These cells inevitably die. The second group of cells, resulting from IR damage, is potentially vital but with a significantly increased risk of cell death. This second group represents cells that we can influence therapeutically (Figure 1).

## 2. Current Therapeutic Options for Influencing IR Damage

In the case of acute ischemic damage, it is important from a clinical point of view to shorten the duration of the ischemia as much as possible, correct the metabolic acidosis, and reduce the risk of acute renal failure and systemic inflammatory reaction (SIRS).

Currently, many experimental and clinical methodologies have been described to reduce or eliminate the extent of the IR damage. Some substances or procedures can only be used to prevent ischemia, while others can only be used to reduce the extent of the IR damage.

A list of options to reduce the range of IR damage in different ways follows:

- A NO (Nitric Oxide) protective strategy [8];
- Adenosine [9];
- The influence of nuclear transcription factors [10];
- The inhibition of apoptosis [11];
- The inhibition of Ca ^2+^ excess in the cell [12];
- Antioxidants [13,14];
- Inhibitors of Na^+^ H^+^ channels [15];
- Controlled reperfusion/reoxygenation [16];
- Intermittent ischemia [17];
- The depletion of neutrophil cells [18,19];
- Aprotinin [20];
- Poly (ADP-ribose) polymerase (PARP inhibitors) [21];
- The blockade of the complement system [22];
- The use of anesthetics [23,24];
- Hypothermia [25,26];
- Hyperbaric oxygen therapy [27];
- MicroRNA as a therapeutic target [28,29];
- Mesenchymal stromal cells therapy [30,31];
- Ischemic tolerance (conditioning).

Most of the research in the field of IR damage is devoted to research on ischemia of the heart and brain. Despite the enormous progress in the understanding of IR damage, there has been no established clinically standard targeted therapy that leads in practice to an unequivocal suppression of IR damage. The problem is the lack of knowledge of the complex multifactorial damage in IR, while most treatment attempts are devoted to the blockade of only a certain mechanism of IR damage. The transfer of knowledge from experimental animals to humans is also problematic because it does not consider the complexity of the ischemic damage, the age of patients, or the comorbidity and co-medication of patients, which can significantly affect the treatment of ischemia.

## 3. Ischemic Tolerance

The use of ischemic tolerance seems to be a reproducible methodology that can adequately protect the organism from IR damage. Ischemic tolerance represents a robust internal defense mechanism and a state in which the cells are resistant to the devastating effect of ischemia, which would lead to cell death [32]. This is one of the forms of evolutionary adaptability [33]. Cells exposed to metabolic stress or sublethal ischemia (preconditioning) become temporarily resistant to the action of the subsequent lethal stress.

The power of protection obtained by ischemic tolerance is surprisingly large. Five minutes of global brain ischemia in rats killed nearly 40% of the most sensitive cells in the brain (CA1 of the hippocampus). A ten-minute global ischemia of the brain led to the death of up to 70% of these cells. The combination of two stresses, either as the action of smaller ischemia first (preconditioning), or, on the contrary, stronger ischemia first with the subsequent action of weaker ischemia (postconditioning), did not lead to a cumulative destructive effect and cell death but to the rescue of these cells [34].

The decisive factor for the activation of ischemic tolerance is the combination of two ischemic or metabolic stresses [34,35]. Activation of ischemic tolerance is a two-step process. The first stress is necessary for the initiation of ischemic tolerance, but for the activation of complete ischemic tolerance, the action of the second stress is absolutely necessary. The advantage is that these two stresses do not have to be of the same nature (cross tolerance), and it is also not important whether they affect the whole organism or only locally (distant tolerance).

**Figure 2:**
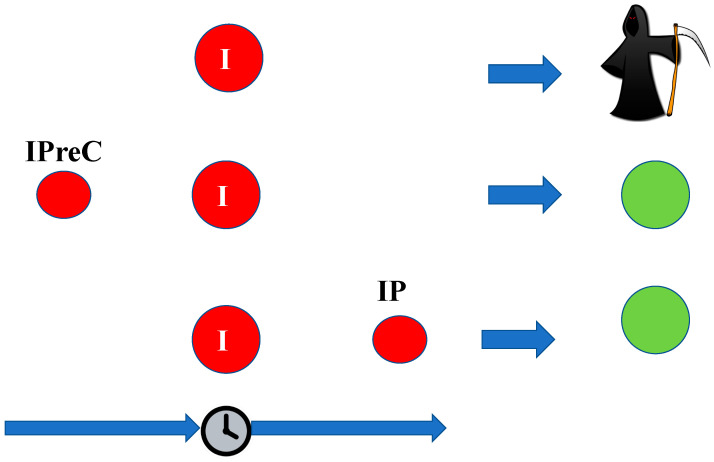
A schematic representation of the different ways of initiating ischemic tolerance. According to the time in which the sublethal ischemia acts relative to the lethal ischemia, we can distinguish the following mechanisms that lead to the initiation of ischemic tolerance. I—ischemic damage, IPreC—ischemic preconditioning, IP—ischemic postconditioning.

According to the time in which the sublethal ischemia acts relative to the lethal ischemia, we can distinguish the following mechanisms that lead to the initiation of ischemic tolerance (Figure 2):

1. Ischemic preconditioning (IpreC) refers to the condition where the sublethal ischemia acts before the lethal ischemia itself;
2. Ischemic perconditioning refers to the condition where the sublethal ischemia and the lethal ischemia act simultaneously;
3. Ischemic postconditioning (IP) refers to the condition when the sublethal ischemia occurs after the lethal ischemia.

## 4. Cross Tolerance

Cross tolerance allows sublethal ischemia in any conditioning modality to be replaced by any one of many biological, physical, or chemical stressors.

Many stimuli or a combination of different stresses can lead to the activation of ischemic tolerance. Cross tolerance works both in the case of preconditioning and postconditioning.

Preconditioning can be induced by stimuli other than ischemia; for example, hypothermia, hyperthermia, hypoxia or hyperoxia, electroacupuncture, or exercise are referred to as cross preconditioning [33].

### 4.1. Hyperoxic and Hypoxic Preconditioning

Normobaric hyperoxia (95% O2) in rats reduced the extent of the cerebral infarct size [36]. The induction of tolerance in this case followed the path of genetic reprogramming [37].

Hyperbaric oxygenation (HBO) indicated tolerance by reducing cell apoptosis and suppressing COX-2 expression in global brain ischemia in rats [36].

### 4.2. Preconditioning Induced by Hyperthermia and Hypothermia

Hypothermia as preconditioning can activate rapidly (early tolerance); however, prolonging the duration of hypothermia does not significantly increase the neuroprotective effect. Instead, increasing the degree of hypothermia is more important [38]. Prolonged ischemic tolerance in this case probably acts through the proteosynthesis pathway.

Hyperthermia can also stimulate tolerance. Hyperthermia of young rats using a warm bath (41.5–42 °C) reduced neuronal damage after an ischemic insult 2 h after hyperthermia [39].

In human liver transplantation, livers were either conventionally cold stored or cold stored and subsequently treated by 1–2 h hypothermic oxygenated perfusion (HOPE) before implantation. Exploratory findings suggest that HOPE decreases the risk of severe liver graft-related events (fewer severe liver graft-related complications and a lower risk of liver-related graft loss) within a 12-month period after liver transplantation. The HOPE approach can be applied easily after organ transport during recipient hepatectomy [40]. Additionally, extracellular vesicles (EV) derived from mesenchymal stromal cells delivered during HOPE can be considered a new organ preservation strategy for increasing the donor pool and improving the transplant outcome because the gluconeogenesis system in HOPE+EV group was preserved [41].

### 4.3. Chemical/Pharmacological Preconditioning

Isoflurane, halothane, and other inhalation anesthetics lead to the activation of ischemic tolerance; they act as antagonists of the NMDA and AMPA receptors [42].

Sevoflurane reduced the extent of the infarct focus and improved the coordination of experimental animals after ischemia. Sevoflurane might exert beneficial effects on cerebral IR-induced neuronal injury through inhibiting the surface expression of the glutamate receptor 1 and blocking CP-AMPAR (calcium permeble-amino-3-hydroxy-5-methyl-4-isoxazolepropionic acid receptor). Halothane is hepatotoxic; therefore, its clinical utility is limited, despite the eventual neuroprotective effect [43,44].

Low doses of liposaccharides can also activate ischemic tolerance in the rat brain [45]. Adenosine receptor agonists can also promote the release of adenosine and the activation of ATP-sensitive K^+^ channels, leading to the activation of ischemic tolerance [46]. A similar effect of pharmacological preconditioning can be achieved by 3-nitropropionic acid [34,47] but also by the application of bradykinin [48] and metformin [49].

### 4.4. Electroacupuncture

Continuous application of electroacupuncture can also act as adequate stress to induce preconditioning [50]. Physical activity, exercise, and transcranial light stimulation [51,52] can also act as adequate stress to induce preconditioning.

### 4.5. Multipotent Adult Progenitor Cells and Tissue Engineering

Normothermic machine perfusion (NMP) of donor kidneys prior to their transplantation provides a good platform for the direct delivery of cellular therapeutics to optimize the organ quality prior to transplantation.

Multipotent Adult Progenitor Cells (MAPC^®^) possess potent immunomodulatory properties that could minimize ischemia–reperfusion injury. Kidneys treated with MAPC cells demonstrated improvement in the clinically relevant parameters and injury biomarkers. This novel method of cell therapy delivery provides an exciting opportunity to recondition organs prior to transplantation [53].

Kidney conditioning with mesenchymal stromal cell (MSCs)/extracellular vesicle (EVs) delivery during hypothermic perfusions protected against ischemic damage by activating the CD73/ADO system [54].

The new strategy of including trimetazidine, tacrolimus, and carvedilol would allow us to shift from cold storage solutions to cold preservation solutions including multitarget pharmacological components, offering protection against IRI and thus protecting the more vulnerable organs [6,55,56].

Tissue engineering offers a promising toolset to tackle ischemia–reperfusion injuries. It devises tissue mimetics by using the unique therapeutic features of stem cells, functional biomaterials, and bioprocesses to design tools to emulate the macroscopic environment that interacts with tissues. This strategy allows the production of cell therapeutics capable of addressing ischemia–reperfusion injury [57].

## 5. Remote Conditioning

As in the case of preconditioning, postconditioning can also lead to the activation of the phenomenon of ischemic tolerance by an attack of sublethal ischemia in a distant organ. Kerendi described for the first time the possibility of postconditioning by clamping the renal artery after 30 min of cardiac artery occlusion. This mechanism of interorgan postconditioning is referred to as remote postconditioning (RP) [58]. It is the application of temporary and short-lasting ischemia on a remote organ (for example, a limb), which can activate resistance to the IR damage on the target organ or tissue.

## 6. Mechanism of the Operation of Local Ischemic Preconditioning

Ischemic preconditioning includes a complex cascade of mechanisms, while their induction also depends on the length of the acting ischemia. Several pathways by which preconditioning can be activated have been described.

### 6.1. Hypoxia-Inducible Factor (HIF-1α)

Hypoxia triggers the production of HIF-1α, which is an oxygen-dependent transcription factor. IPreC has a different effect on the expression of HIF-1α in different cells; its expression in neurons is fast, while in astrocytes it is slower but continuous [59,60].

HIF-1α is also formed in skeletal muscle after ischemia and during preconditioning through the transcription factor EGR1 (Early Growth Response 1) and the expression of cFOS genes, but it suppresses the expression of cJUN genes [61].

The formation of HIF-1α clearly promotes the expression of endothelial growth factor (VEGF) and the activation of nuclear factor kappa B (NF-κB), which can attenuate neuronal death induced by ischemic attack even in the hippocampus CA1 region, which is the brain region most sensitive to the influence of ischemia [62].

### 6.2. Glutamate Pathway

This pathway of the activation of ischemic tolerance is based on excitotoxicity glutamate. After the action of ischemia, there is a decrease in the intracellular ATP reserves, while at the same time there is an excessive accumulation of glutamate, which stimulates the N-methyl-D-aspartate (NMDA) receptor, which results in an excessive influx of calcium into the cell, damage to synaptic plasticity, and the accumulation of glutamate [63].

Without adequate activation of the NDMA receptors, the activation of ischemic tolerance will not occur; this activation mechanism includes the NF-κB and the tumor necrosis factor-α (TNF-α) pathway [64].

IPreC can block gap junctions (connections between astrocytes where connexin 43 is present), reduce extracellular glutamate, and reduce ROS in astrocytes, all of which lead to less damage to neurons [65].

### 6.3. NO Oxide Synthase (NOS)

NO is important in the mechanism of IPreC activation, but the exact mechanism of its action is still unknown. With the application of the NOS inhibitor (7-nitroindazole) after IPReC, the activation of ischemic tolerance was stopped [66].

eNO synthase is considered to protect against vascular spasm caused by a subarachnoid hemorrhage; that is, NOS is involved in the activation of ischemic tolerance by several mechanisms [66].

### 6.4. Pathway of the CD39-CD73-Adenosine Receptor

Adenosine is an antithrombotic agent, which protects against oxidative stress and suppresses the immune response. Evidently, therapies that promote adenosine generation or boost CD39 activity at the site of endothelial injury have promising benefits in the context of atherothrombotic stroke and can be extended to the current cerebral small-vessel disease’s known pathomechanisms [67], but its effect was also confirmed in the heart and liver, as mentioned below.

CD39 and CD73 (two of the ectonucleotidases) are involved in converting ATP into extracellular adenosine, which is the key metabolite that accumulates and inhibits the function of important immune cells, including T cells and NK cells, leading to an environment conducive to tumor growth. Consequently, multiple clinical strategies are being explored to target this pathway for the treatment not only of cancer, but also IR injury.

Tissue protection is ensured by CD73 enzyme-dependent adenosine generation, and it is signaled through the adenosine-mediated ADORA2B receptor, which triggers tissue protection. Moreover, the adenosine A2B receptor agonists may be used as a potential preventive therapy against IR injury in flap surgeries [68].

Hepatic ischemia should be also tempered with preconditioning, which is associated with the significant induction of the CD39 transcript, heightened protein expression, and improved outcomes after IR injury. Moreover, mimicking ischemic preconditioning with i.p. apyrase (a soluble ectonucleoside triphosphate diphosphohydrolase, NTPDase) in the absence of preconditioning attenuated hepatic injury after IR injury [69].

Ecto-5′-nucleotidase (CD73), the “pacemaker” enzyme of extracellular adenosine production, plays an important role in protection. Moreover, the use of soluble 5′-nucleotidase may be a potential therapeutic for hepatic ischemia [70]. The effectiveness of ecto-5′-nucleotidase (CD73) is ensured by the activation of cell-surface adenosine receptors (A_1_AR, A_2A_AR, A_2B_AR, A_3_AR); it is effective not only in the liver but also in cases of myocardial ischemia; so, 5′-nucleotidase or A_2B_AR agonists can be used as therapy for myocardial ischemia [71]. Ischemic preconditioning increases the level of CD39 in the heart and contributes to cardiac protection. Cardiac-specific expression of CD39 reduced myocardial dysfunction and infarct size following ischemia–reperfusion injury [72].

The production of anti-inflammatory adenosine reduces the donor kidney antigenic load, and these strategies may translate to improved transplant survival. CD39 in the vascular endothelium and in circulating cells, in particular, regulatory T cells (Treg), is upregulated in response to hypoxic stimuli and plays a critical role in regulating the immune response removing the proinflammatory ATP [73].

Administration of sesame could effectively protect kidney from IR injury by inhibiting inflammatory responses, which might be associated with promoting the adenosine–CD39–A2AR signaling pathway [74].

### 6.5. Immune Pathway

During ischemia, large numbers of inflammatory cells (such as microglia, lymphocytes, and neutrophils) migrate to the site of infarction (ischemia), and these cells mediate inflammatory brain damage [75].

The noncatalytic Toll-like (TLR) receptor pathway can induce the transcription factor NF-κB, which mediates the transcription of cytokines and chemokines. These are responsible for the initiation of the immune response and the formation of the inflammatory cascade.

IPreC itself provides neuroprotection by promoting an anti-inflammatory response by inhibiting the TLR4/NF-κB signaling system [76].

### 6.6. Enzymes and Receptors

The natural function of cyclooxygenase-2 (COX-2) is to assist in the oxidation of arachidonic acid to the prostaglandin, which has an important role in inflammatory damage after ischemia. IPreC led to the suppression of COX-2 formation in gerbils, thereby suppressing inflammatory manifestations in the ischemic brain lesion [77].

An increase in adenosine can be observed after brain ischemia; however, after IpreC, an increased expression of adenosine receptors has also been observed [78]. It is also assumed that adenosine receptor A1 is involved in the activation of ischemic tolerance. Adenosine kinase (ADK) inhibits the formation of adenosine, and its eventual suppression in the brain can lead to neuroprotection [79].

During reperfusion in the brain, unbound proteins accumulate in the endoplasmic reticulum. IPreC can inhibit stress-induced apoptosis, thereby contributing to the activation of tolerance [80].

### 6.7. Autophagy and Apoptosis

Autophagy is the process of internalizing and “digesting” one’s own damaged organelles. This process is involved in IPreC mechanisms. Ischemic injury activates autophagy [81]. Interestingly, significant autophagy activation appeared after three IPreC cycles of 5 min [82].

Conversely, apoptosis as a result of IR damage works mainly on a caspase-3-dependent pathway. Ischemia leads to overexpression of caspase-3, but IPreC can suppress this expression, resulting in the formation of ischemic tolerance [83]. Induction of autophagy appears to be a possible method of activating ischemic tolerance.

### 6.8. Energy Metabolism

During brain ischemia, there is a change in the energy metabolism from oxidative phosphorylation to excessive glycolysis, which allows the generation of ATP to cover the energy needs.

Excessive glycolysis increases the concentration of lactate and ROS, further promoting the expansion of ischemic changes. IPreC suppresses postischemic hyperglycolysis and promotes the utilization of β-hydroxybutyrate, thereby normalizing energy metabolism [84].

### 6.9. Permeability of the Blood–Brain Barrier

As a result of ischemia, there is a breakdown of the blood–brain barrier, which allows the infiltration of immune cells into the site of the ischemia [85]. IPreC can preserve the paracellular permeability of the membrane by supporting the formation of claudins and adherins, which are part of the tight and adherent connections of this membrane [86].

### 6.10. Transcriptional Regulation

Some works point to the importance of the transcription of survival genes after ischemia.

After hypoxic stress, HIF-1α and HIF-1β join together to form a HIF-1 heterodimer, which binds to the target genes, forming a transcriptional complex enabling the production of VEGF, erythropoetin, and glucose transporters [87].

### 6.11. Genetic Reprogramming

IPreC causes not only an increased expression of genes associated with cell protection but also a simultaneous suppression of the expression of the genes associated with cell degeneration after ischemic injury. Heat shock protein 72 (HSP-72) [88] and transforming growth factor (TGF-A) [89] are thought to be involved in this reprogramming.

### 6.12. Epigenetic Reprogramming

MicroRNA (miRNA) also has an important role in mediating the effect of IPreC. Lethal ischemia itself leads to suppression of miRNA transcription; in contrast, IPreC promotes its creation [90].

### 6.13. Activation of Ischemic Tolerance in the Brain

In an experimental model, the pre-exposure of animals to an enriched environment led to the activation of preconditioning. The major role was played by the activity-dependent transcription factor Npas4, whose expression is triggered by excessive Ca^2+^ influx. Furthermore, Npas4 regulates L-type voltage-gated Ca^2+^ channels through expression of the small Race-like GTPase Gem in ischemic neurons [91].

Preconditioning also initiates glial responses, especially in astrocytes, which transforms them into an ischemia-resistant phenotype. P2X7 receptors (P2X7Rs) in astrocytes play a main role in preconditioning; although P2X7Rs usually trigger inflammatory and toxic responses, preconditioning triggers P2X7Rs in astrocytes to function as a switch to protect the brain against ischemia [47,92].

However, because inhibitors of astrocytes activation abolish ischemic tolerance, it is interesting to consider the possibility that the neuron-dependent ischemic tolerance acquisition mechanism is not neuron-autonomous but rather neuron-non-autonomous as a result of communication with the glial cells. It should be noted that apart from microglia and astocytes, the vascular system and oligodendrocytes are involved in the activation of ischemic tolerance [93].

## 7. The Mechanism of Remote Ischemic Preconditioning

As of now, there has been no clearly confirmed method of propagating the initiation of ischemic tolerance by the body. Three theories of the spread of the triggers of ischemic tolerance are known: humoral, nervous, and immune. Following the transport of tolerance triggers to the target organ, the initiation of a common signaling pathway is predicted.

### 7.1. Humoral Pathway

The assumption of the existence of a humoral pathway is confirmed by two further assumptions. First, after preconditioning, a period of reperfusion is required, meaning that the tolerance triggers must be blood-borne before reaching the target organ. Secondly, in models of the cross-administration of activated blood or plasma, not only intergeneric but also interspecies transfer of tolerance triggers were proven. Shimizu described the possibility of transferring activated plasma (limb ischemia) to other individuals, and only by administering this plasma did the activation of ischemic tolerance and protection of the myocardium from subsequent ischemic damage occur. Based on this study, it can be assumed that the triggers of ischemic tolerance were <15 kDa molecules. The activated plasma effect can be blocked by naloxone’s blockers; so, it should be expected that opioid receptors will be involved in the activation of tolerance, and the functioning of the transfer of the tolerance triggers will be independent of the nervous system [94]. Based on other works, it can be assumed that interleukins, stromal factor+-α, TNF-α, bradykinin-2, adenosine, opioids, NO, miRNA, and possibly catecholamines can be possible triggers of tolerance [95]. The assumption of humoral transfer clearly supports the transfer of ischemic tolerance with blood plasma from a 2× conditioned donor [96,97].

### 7.2. Nerve Pathway

Administration of the ganglia blocker (hexamethonium) leads to the cessation of the activation of ischemic tolerance after the application of limb RIPreC; therefore, it can be concluded that the nerve pathway is also involved in the transmission of tolerance triggers [98].

The activation of dorsal neurons in the brainstem (vagal preganglionic neurons) produces a similar effect to the application of RIPreC. Moreover, by activating parasympathetic efferent nerves, neuroprotection will be activated, and the size of the infarcted focus will decrease [99,100].

It has been described in animal models that after transection of the femoral nerve, possibly the spinal cord, the RIPreC effect was abolished [101]. The neuroprotective effect of RIPreC on mice was only partially suppressed, when the femoral or sciatic nerve was damaged; so, it is necessary to think about the simultaneous connection of the humoral and nerve activation during the transfer of tolerance triggers [56,101].

Transection of the femoral nerve in an animal model stopped the activation of tolerance after limb RIPreC, but electrical stimulation of the damaged femoral nerve restored this activation of tolerance [102].

The release of endogenous humoral factors from the conditioned organ probably first activates the afferent nerve fibers in the conditioned organ and subsequently activates the efferent nerve fibers leading to the target organ.

Moreover, in patients with peripheral neuropathy, the RIPreC effect was weakened and even suppressed, which points to the involvement of the nerve pathway [99,103].

### 7.3. Inflammatory Pathway

Limb RIPreC inhibits inflammatory changes. It suppresses proinflammatory genes and stimulates anti-inflammatory genes. The influence of the genes is manifested by the reduction in the number of circulating neutrophils and the inhibition of the release of cytokines. This effect is already noticeable 24 h after the action of RIPreC [104,105].

The activation of immune cells and the regulation of inflammatory genes are associated with the release of endogenous opioids; therefore, the humoral and immunological pathways of activation will also be interconnected [106].

### 7.4. Common Final Mechanisms

Based on the previous results, it can be concluded that in the transmission of ischemic tolerance triggers, there is a mutual combination of their transmission via the humoral, nervous, and immunological pathways. After their transfer to the target organ, the common path of the activation of tolerance itself is activated. Upon activation of the tolerance in the target organ after the arrival of triggers, autophagy is activated, which promotes the degradation of the damaged cell organelles and accumulated proteins. Limb IRPreC stimulates autophagy and supports the emergence of neuroprotection [107]. Mitochondria also participate in this common mechanism of tolerance activation; the action of RIPreC supports the preservation of the structural and functional integrity of mitochondria, the reduction in mitochondrial degradation and, consequently, the reduction in apoptosis [108].

## 8. Cell Signaling Pathways in Ischemic Postconditioning

### 8.1. Akt Signaling Pathway Activation

The PI3K/Akt is a cellular pathway important in cell cycle regulation that regulates cell survival by inhibiting apoptosis and cell growth [109].

IP leads to an improvement and prolongation of Akt phosphorylation, which ultimately leads to a reduction in the extent of the necrosis at the ischemic site [110].

In the case of RIP, this pathway also applies, leading to a reduction in mitochondrial damage [110].

### 8.2. The mTOR Signaling Pathway of Activation (Mammalian Target of Rapamycin = Thr/Ser Protein Kinase)

The mTOR pathway plays a major role in cell metabolism, growth, differentiation, development, and cell survival [111]. However, its role in IR damage is controversial. mTOR is inhibited by hypoxia and the lack of ATP; in contrast, antioxidants, melatonin, and estradiol stimulate and affect this pathway [112].

In IP, blocking the mTOR pathway using its inhibitor rapamycin cancels the protective effect of IP [113,114].

### 8.3. The Mitogen-Activated Protein Kinase (MAPK) Pathway

MAPK is a major kinase pathway that transduces extracellular signals into an appropriate cellular response. As with the mTOR pathway, this pathway also mediates cell growth, development, division, and differentiation.

IP increases the expression of ERK1/2 kinase and activates the MAPK pathway; however, after the application of kinase blockers, the inhibition of the neuroprotective effect does not occur, as would be expected; therefore, the role of the MAPK pathway is rather unclear in cooperation with postconditioning [110].

### 8.4. The Protein Kinase C (PKC) Pathway

PKC is a serine/threonine protein kinase that has at least 12 isozymes. IP blocks cell death by promoting εPKC phosphorylation [115]. δPKC activity promotes cell death, but εPKC activity promotes neuronal survival [116].

### 8.5. The Toll-Like Receptor 4 (TLR4) Pathway

The TLR4 pathway is an important mediator of the immune response that leads to inflammation and is part of the activation of ischemic tolerance. Both IP and RIP lead to suppression of the TLR4/NF-κB pathway [117,118].

In the brain, the mechanism causing apoptosis of the CA1 neurons in the hippocampus also plays an important role. After ischemia, despite restoration of energy metabolism, there is no restoration of protein synthesis. That is caused by phosphorylation of the small α subunit of initiation factor 2 (eIF2α). The phosphorylation of eIF2α causes inhibition of the translation initiation with the subsequent disintegration of the polyribosomes into subunits [119].

If protein synthesis is not restored, the cells die by so called delayed death of neurons. Postconditioning allows the unblocking of proteosynthesis [88,120].

### 8.6. The Mitochondrial Role in Postconditioning

Mitochondria play a critical role in IR injury and the subsequent development of ventricular systolic dysfunction and possible compensatory heart hypertrophy [121].

The mitochondrial permeability transition pore (mPTP) has a pivotal influence on cells’ ability to survive or die. Postconditioning can block the opening of mPTP, which has detrimental effect on the activation of a robust anti-ischemic protection. Mitochondrial calcium is decreased during preconditioning; in contrast, it is increased significantly either in postconditioning or after the inhibition of mPTP [122,123]. Calcium changes in assembly explain why reperfusion injuries are diminished in postconditioned hearts. Postconditioning itself is attributed to the opening of the mitochondrial KATP channels (mKATP), and the inhibition of the mPTP opening leads to a reduced infarct size [124].

It is also expected that the activator of transcription 3 (STAT3) contributes to cardioprotection by the stimulation of respiration and the inhibition of the mPTP opening [125]. Moreover, mitochondrial ATP-sensitive K+ channels (mKATP) and connexin 43 have tasks in cardioprotection in postconditioning [126,127].

Mitochondria influence H_2_O_2_ production and redox stress during reperfusion through mKATP activation [128,129]. Moreover, phosphatidylinositol 3-kinase (PI3K) is involved in the regulation of mPTP [130].

Hydrogen sulfide (H_2_S), carbon monoxide (CO), and nitric oxide (NO) are recognized as three gaseous mediators for cardioprotection, which should also be produced within mitochondria [131,132]. Anesthetics (isoflurane, sevoflurane, and propofol) are also agents targeting mitochondria with prominent postconditioning effects [133,134,135].

Mitochondria are important players in many types of apoptotic and necrotic cell death [128]. Postconditioning signaling converges on the mitochondria, and it increases the levels of antiapoptotic markers, including the phospho-GSK-3β and Pim-1 kinases, while decreasing the proapoptotic markers, namely cytochrome c, thus preserving the mitochondrial morphology [136,137].

## 9. Pharmacological Postconditioning

The mechanism of ischemic postconditioning includes activation of the adenosine receptors, α-adrenergic receptors, opioid receptors, and bradykinin, with activation of pathways such as the PKC, TK, and MAP kinase, or the activation mechanism involves an endogenous trigger such as NO and Ca^2+^ ions. The administration of drugs whose effect mimics the mechanism of the action of ischemic postconditioning is called pharmacological postconditioning (FAP) [138,139].

FAP has been described in the heart, brain, and liver. Different mechanisms and receptors are involved in its functioning, while the protection obtained by FAP is based on inflammatory, antiapoptotic, and antioxidant processes. The advantage of FAP over IP is the possibility of its application even at a time when IP cannot be used, for example due to the impassability of a local artery in the basin of which ischemia has occurred.

The mechanism of FAP is direct interference with the course of the path of the lethal damage or the induction of a low-threshold stress, which can trigger the activation of tolerance against a stronger, i.e., lethal stress [140].

For example, the application of isoflurane after 60 min of global brain ischemia reduced the extent of brain damage in both in vivo and vitro models [141].

Moreover, the application of morphine, which leads to the stimulation of opioid receptors, can activate neuroprotection after the end of lethal ischemia, similar to ischemic postconditioning [142].

## 10. Ischemic Tolerance (Closing Remarks)—Application Possibilities

Even though the phenomenon of ischemic tolerance has been known for more than 30 years, we still only have partial information about its activation and functioning. So far, there is a lack of a comprehensive understanding not only of the mechanisms of its functioning but also of how to adequately transform the experimental results into real clinical use.

Individual tissues have significantly different resistance to IR damage. This resistance is partially dependent on the energy metabolism of the cells but is also not fully understood. In the brain, cells in the CA1 hippocampus are the most sensitive to ischemia, which begin to die after 5 min of global cerebral ischemia. Ten minutes of global ischemia of the rat brain led to the death of up to 70% of these cells [143]. Conversely, in the case of limbs, skeletal muscle is significantly more vulnerable than peripheral nervous tissue. In skeletal muscle, necrotic changes appeared after 2–3 h of ischemia, but in the peripheral nerve, signs of nerve cell degeneration did not appear until after 5 h of ischemia [144]. This different sensitivity of cells to IR damage must also be considered when investigating ischemic tolerance.

Until now, the clinical use of ischemic tolerance was only preconditioning. In this case, the application of sublethal stress activates temporary ischemic tolerance to stresses of different genesis, which allows survival under other circumstances of lethal stress.

This classic preconditioning is applicable only in the case of elective surgical interventions, during which ischemia of the organism or its part or organ is expected in advance, for example during the transfer of free lobes, elective replantations, revascularization, or operations such as aortocoronary bypass.

The preconditioning itself must be applied before the ischemia of the organ occurs, i.e., at the latest at the beginning of the revascularization operation, for example, before the ischemia occurring during the suture of the distal vascular anastomosis during the suture of the graft to the coronary vessel [145].

The disadvantage of local preconditioning is the one-time or repeated clamping of the vascular pedicle of the lobe, which can lead to intimal damage to the supply artery of the lobe. This problem also applies to local postconditioning.

The limited applicability of preconditioning lies in the fact that it is not always possible to predict the onset of ischemic damage. This fact significantly limits the applicability of preconditioning in clinical practice. Unfortunately, the exception is a war conflict, where it is possible to assume the occurrence of damage, the consequences of which could be significantly reduced using preconditioning in young, healthy, and unmedicated people.

From a clinical point of view, postconditioning has a much greater application due to the frequent unexpected occurrence of ischemia (for example, amputation of fingers, thrombosis of vascular pedicles of free lobes, damage to soft tissues in fractures of the proximal and distal tibia) and many other life, organ, or limb threatening conditions. The applicability of this methodology after exposure to lethal stress is an indisputable advantage.

The application of postconditioning is more useful in clinical practice, as it allows the living organism to activate the mechanism of ischemic tolerance, which can reverse the process of cell degeneration even within a few hours after the occurrence of lethal stress. It is necessary to emphasize that despite the fact that five or more minutes of brain ischemia in humans represents the beginning of irreversible changes, neurons undergo apoptosis similar to the so-called delayed death of neurons, and the therapeutic window of their rescue is significantly longer than in other types of cell death.

The use of short-term skeletal muscle ischemia (delayed postconditioning) can reverse the death of muscle cells after 3 h of ischemia and also prevent muscle edema [146]. These results correspond with those of Tsubota [147], who applied remote postconditioning to the contralateral limb in mice. In the case of remote postconditioning, the setting of an adequately large ischemia (its length and intensity) necessary to activate ischemic tolerance is still an open question. The application of ischemia to the upper limb has not been proven in humans, there is a preference for the application of ischemia to the lower limb. The reason is probably the larger amount of skeletal muscle in the lower limb, the ischemia of which leads to a significantly stronger stimulus for the activation of ischemic tolerance. Moreover, the determination of the length of the ischemia time interval has been extensively researched; we ourselves are inclined to 20 min of muscle ischemia, the most effective according to the results of Pignataro [148]. This period of ischemia is not dangerous for animals or humans, during which there is no damage to the vitality of the muscles.

If short-term ischemia of a relatively large skeletal muscle is used as postconditioning, it induces two synergistic beneficial effects [97]:

- Induction of ischemic tolerance;
- Temporary elevation of blood pressure and blood flow.

The effect of a temporary increase in perfusion caused by remote ischemic postconditioning has a positive effect in clinical practice, for example during the transfer of free, rotary flaps, or during finger replantation. Even today, free flaps are a reliable method of covering defects in trauma surgery, but their rejection (complete necrosis) occurs in up to 25% of cases, and partial necrosis is present in up to 36% of cases. Rejection is a consequence of the insufficient perfusion of the lobe. In addition, postconditioning-induced postischemic reactive hyperemia can act as a prevention of edema, the “no reflow” phenomenon, and an overall improvement in local microcirculation [149].

Although the use of remote ischemic local postconditioning is an effective method in properly selected cases, its universal clinical application is rather complex (tourniquet loading).

Despite the very encouraging and successful results in animal experiments, the clinical results in humans have been clearly disappointing so far. Most of the human studies concerned the use of the phenomenon of ischemic tolerance to reduce the extent of heart attack and improve the biochemical parameters [150,151,152]. Ultimately, RIPreC did not reduce the number of serious cardiac and cerebrovascular complications for cardiac surgery. Adequate protection of the myocardium has also not been demonstrated.

Due to the abovementioned facts, it will be clearly necessary to transfer the results from young healthy animals to the gerontic population, which suffers from numerous comorbidities (diabetes mellitus, hypertension, and hyperlipidemia) and co-medications (antioxidants, statins, β-blockers, ACE inhibitors, AT1 receptor antagonists, antagonists Ca channels, and nitrates) that block the emergence of ischemic tolerance [150,153,154]. Comorbidities clearly lead to the suppression of ischemic tolerance [155].

Since the effectiveness of remote ischemic postconditioning was also proven in the case of delayed neuronal death (“apoptosis”) induced by temporary global ischemia of the brain or kainate intoxication, it can be assumed that conditioning products are able to overcome the blood–brain barrier [156], i.e., they are probably evenly spread throughout the body.

Based on Shimizu’s results, it can be assumed that triggers of ischemic tolerance are contained in plasma, are low molecular hydrophobic substances, are independent of local neurogenic activity, and require the activation of local opioid receptors [94]. The idea of transferring these substances between individuals of the same species but also between species is revolutionary. However, even with the transfer of triggers from the donor, the activation of tolerance may be blocked (possible comorbidity and comedication).

The effect of preconditioned plasma was also demonstrated by Zhao [157]. Preconditioned plasma (taken from donor animals 48 h after limb ischemia) was able to reduce the extent of IR damage to the myocardium after transfer to recipients, which was clinically manifested by a reduction in the incidence and duration of ventricular tachycardia.

A similar result was also achieved by Weber [158]. By applying ischemic preconditioned plasma in volunteers, he achieved protection of human umbilical endothelial cells from damage indicated by hypoxia. In this case, the plasma was collected directly after the preconditioning of the volunteers.

In all cases mentioned, it was only a transfer of “partially” activated plasma. If the plasma was activated by only one stress, only triggers of ischemic tolerance were present in it. In our experiments, twice-activated plasma was used (exposure to 2 stresses in a period of 48 h, with plasma withdrawal 6 h after the second stress). Withdrawal of doubly activated plasma involves the transfer of effectors, not triggers, of ischemic tolerance. The use of a proteosynthesis inhibitor (cycloheximide), which blocked the emergence of tolerance, serves as proof of the necessity of synthesizing protein effectors [35].

Our results clearly confirmed the efficacy of doubly activated plasma when administered to recipient animals after lethal ischemia. The advantage of doubly activated plasma containing complete tolerance also lies in the fact that co-medication and co-morbidity in the recipient should not have an adverse effect on its immediate effectiveness, which will allow its easy application and immediate effectiveness.

Until there is a clear chemical identification of the end products (end effectors) of ischemic tolerance, the simplest way to activate ischemic tolerance will be the preparation of activated plasma from young healthy donors with the possibility of its immediate use in recipients during the initial treatment.

The next expected stage is the exact identification of the effector (product) of ischemic tolerance and its direct administration to the patient. Based on several works [36,97], it can be concluded that the end effector of ischemic tolerance passes through the blood–brain barrier, meaning that its application will not be limited to only one organ, and its action will be possible immediately in the whole organism. These assumptions were also confirmed by our results [96,97], where we clearly demonstrated the unequivocal effect of activated plasma on the survival of skeletal muscle cells as well as brain cells in the CA1 hippocampus.

## Data Availability

Not applicable.
